# Dermal fibroblasts and triple-negative mammary epithelial cancer cells differentially stiffen their local matrix

**DOI:** 10.1063/5.0021030

**Published:** 2020-12-04

**Authors:** Alicja Jagiełło, Micah Lim, Elliot Botvinick

**Affiliations:** 1 Department of Biomedical Engineering, University of California Irvine, Irvine, California 92697, USA; 2 Beckman Laser Institute and Medical Clinic, University of California Irvine, Irvine, California 92612, USA; 3 The Edwards Lifesciences Center for Advanced Cardiovascular Technology, University of California Irvine, Irvine, California 92697, USA

## Abstract

The bulk measurement of extracellular matrix (ECM) stiffness is commonly used in mechanobiology. However, past studies by our group show that peri-cellular stiffness is quite heterogeneous and divergent from the bulk. We use optical tweezers active microrheology (AMR) to quantify how two phenotypically distinct migratory cell lines establish dissimilar patterns of peri-cellular stiffness. Dermal fibroblasts (DFs) and triple-negative human breast cancer cells MDA-MB-231 (MDAs) were embedded within type 1 collagen (T1C) hydrogels polymerized at two concentrations: 1.0 mg/ml and 1.5 mg/ml. We found DFs increase the local stiffness of 1.0 mg/ml T1C hydrogels but, surprisingly, do not alter the stiffness of 1.5 mg/ml T1C hydrogels. In contrast, MDAs predominantly do not stiffen T1C hydrogels as compared to cell-free controls. The results suggest that MDAs adapt to the bulk ECM stiffness, while DFs regulate local stiffness to levels they intrinsically prefer. In other experiments, cells were treated with transforming growth factor-β1 (TGF-β1), glucose, or ROCK inhibitor Y27632, which have known effects on DFs and MDAs related to migration, proliferation, and contractility. The results show that TGF-β1 alters stiffness anisotropy, while glucose increases stiffness magnitude around DFs but not MDAs and Y27632 treatment inhibits cell-mediated stiffening. Both cell lines exhibit an elongated morphology and local stiffness anisotropy, where the stiffer axis depends on the cell line, T1C concentration, and treatment. In summary, our findings demonstrate that AMR reveals otherwise masked mechanical properties such as spatial gradients and anisotropy, which are known to affect cell behavior at the macro-scale. The same properties manifest with similar magnitude around single cells.

## I. INTRODUCTION

Bulk stiffness of the extracellular matrix (ECM) has been previously shown to regulate cellular processes and correspond to invasiveness of cancer cells.^1–3^ ECM stiffness is a measure of ECM resistance to deformation and is primarily regulated by ECM remodeling, strain stiffening, degradation, and deposition carried out by cells in response to a variety of biochemical cues.^1^ Factors including aging, genetic mutations, diabetes, and other medical conditions have also been shown to modify mechanical properties of the ECM.^4^ The majority of research related to mechanical aspects of cell-ECM interactions relies on measuring the bulk ECM stiffness as a single parameter^5–7^ or otherwise equating stiffness with the density or concentration of hydrogels or substrates to which cells are exposed.^8,9^ These approaches do not directly measure the stiffness of the peri-cellular region within naturally derived fibrous three-dimensional ECMs, such as those comprising type 1 collagen (T1C) or fibrin. Our laboratory uses optical tweezers active microrheology (AMR) that provides access to the peri-cellular region. In fact, past research in our laboratory has shown that the peri-cellular stiffness on a single cell level can span orders of magnitude.^10^ These findings prompted us to investigate how cells remodel their local stiffness in correlation with bulk (e.g., cell-free) ECM stiffness and other mechanical and biochemical cues.

In this study, we use AMR to measure stiffness around two migratory cell types—highly invasive, triple-negative breast cancer cells MDA-MB-231 (MDAs) and normal human dermal fibroblasts (DFs). While highly migratory and dynamic DFs are key regulators of ECM stiffness and composition,^11,12^ MDAs are thought to be regulated by tissue stiffness, which relates to early screening for breast cancer by detecting elevated breast density and stiffness.^13^ Consequently, measuring stiffness around both cell lines is of scientific interest to the field of mechanobiology. DFs and MDAs were cultured within T1C hydrogels, chosen because T1C is the most abundant component of these cells' ECM ^12^ and is known to regulate cell processes and behaviors.^14,15^ Also, collagens are known to be remodeled and crosslinked during cancer progression.^13–15^

In this study, we assess changes in peri-cellular stiffness of MDAs and DFs in response to (1) human transforming growth factor-β1 (TGF-β1), (2) glucose, and (3) Y27632. These factors were shown to alter cell migration, proliferation, and cell contractility of MDAs and DFs.^16–20^ In cancer cells, TGF-β1 was shown to promote immunosuppression, angiogenesis, and epithelial-mesenchymal transition (EMT), which are primary mechanisms leading to breast cancer metastasis.^13^ Moreover, TGF-β1 was reported to strengthen focal adhesions and result in increased migration of different cancer cell lines, including MDAs.^2,18^ Its effect on cell migration was further correlated with cell invasiveness and metastatic potential.^17,21^ Addition of TGF-β1 also promotes collagen synthesis in DFs and might even result in differentiation of DFs into myofibroblasts under high tensile stresses.^12^ The addition of TGF-β1 to media was, therefore, expected to increase stiffness around both DFs and MDAs.

Elevated concentrations of glucose were reported to promote cancer cell proliferation, a phenomenon attributed to the Warburg effect, which favors aerobic glycolysis over oxidative phosphorylation in cancer cells.^22^ Hyperglycemia additionally lowers survival rates in malignant breast cancer patients and mitigates the efficacy of cancer treatments by promoting chemoresistance and aggressiveness of cancer cells including MDAs,^4,23^ as indicated by their increased proliferation and reduced apoptosis.^24^ We, therefore, assumed that glucose addition to the media would also result in larger peri-cellular stiffness levels. In contrast, the addition of glucose to fibroblasts was previously described to promote collagen deposition but reduce both the proliferation and migration rate of fibroblasts, as commonly observed in delayed wound healing responses in diabetic patients.^25–28^ Nonetheless, despite reduced migratory capabilities of DFs, contractile properties were shown to be increased in fibroblasts cultured in high glucose media as opposed to low glucose media.^28^ Thus, we expected that increased cell contractility should result in elevated peri-cellular stiffness levels as compared to control cells cultured in low glucose media.

Next, we targeted Rho-associated protein kinase (ROCK) that is overexpressed in tumorigenic and metastatic breast cancer cell lines, including MDAs.^19,29^ ROCK is primarily responsible for organizing the cell cytoskeleton and stimulating cancer cell metastasis by increasing focal adhesions and disrupting cell–cell junctions. Consequently, ROCK enhances cell contractility, migration, and proliferation, all of which promote cancer invasiveness.^30^ Inhibition of the ROCK signaling pathway is, hence, expected to prevent strain stiffening of peri-cellular collagen fibers and, consequently, reduce peri-cellular stiffness around MDAs. In our experiments, we use the ROCK inhibitor Y27632, which has widely documented anti-invasive, anti-migratory,^29,31^ and anti-proliferative^9^ properties in breast cancer studies. Previous studies in our laboratory indicate that Y27632 prevents cell contractility and ECM stiffening by DFs^10^ and Y27632 was also expected to yield a similar effect on MDAs.

The AMR results described below demonstrate that both MDAs and DFs can adapt to their environment and modify it in response to a variety of mechanical or biochemical factors, which were previously shown to either promote or reduce cancer cell invasiveness and fibroblast contractility. Unlike bulk stiffness measurements, experiments at the single cell level also allow us to better explain how cell-ECM interactions are spatially dependent on these different treatments and collagen concentrations.

## II. RESULTS

**FIG. 1.**
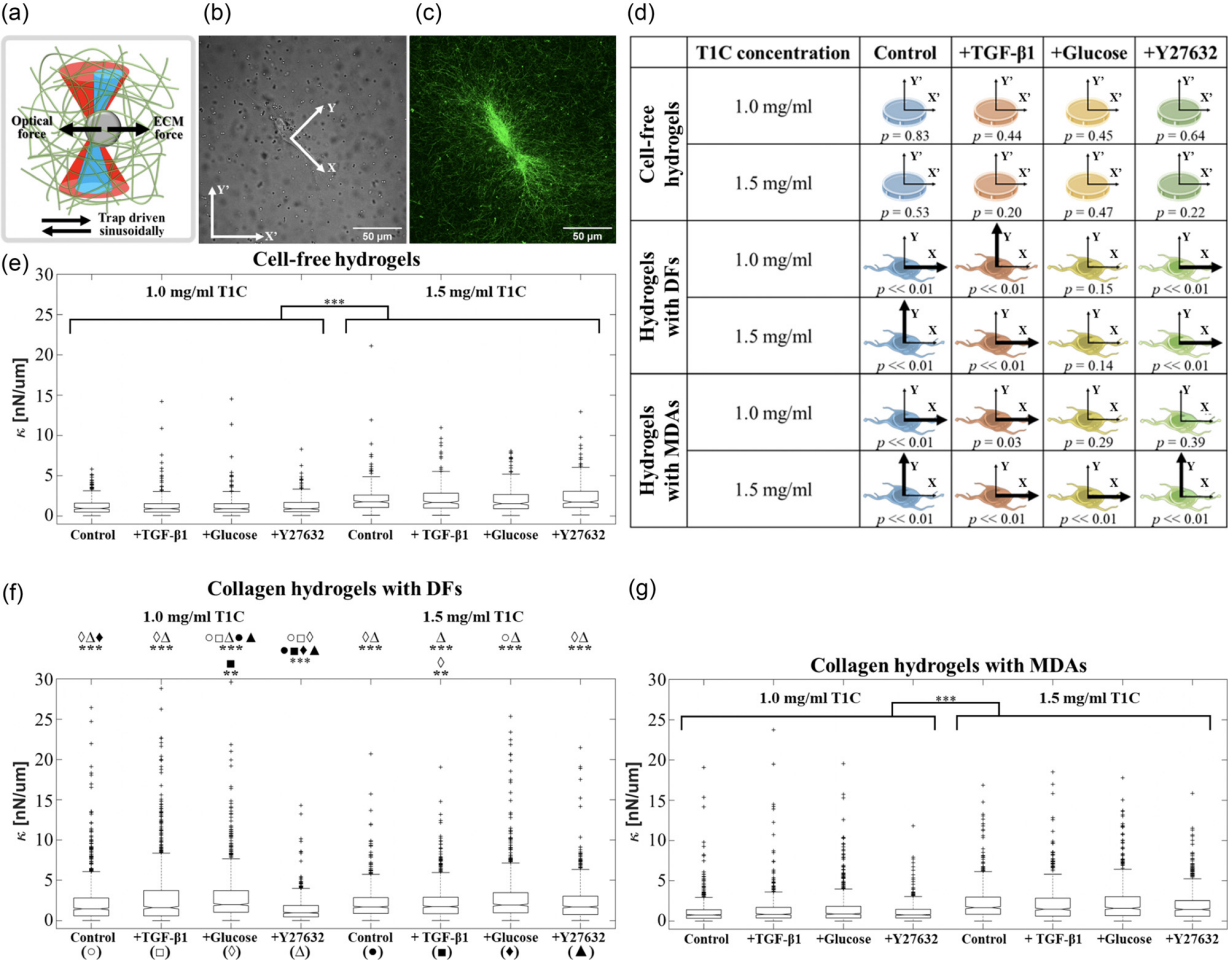
Aggregated stiffness values assessed by optical tweezers active microrheology. (a) Diagram of optical tweezers active microrheology. The optical tweezers beam (red) is oscillated sinusoidally in the horizontal (*X'*) or vertical (*Y'*) direction with respect to the field-of-view. The optical trap exerts oscillatory forces on a probed microbead (gray), while ECM (green) resists the bead displacement. The bead displacement is probed by a stationary detection beam (blue) (details in Fig. 1 of the supplementary material and Methods). (b) Brightfield and (c) reflection confocal microscopy images of a DF embedded in a 1.0T1C hydrogel. Stiffness measured along the *X′* and *Y′* axes is projected onto axes corresponding to the long (*X*) and short (*Y*) axes of the cell with origin at the cell centroid. (d) Graphical representation of stiffness anisotropy. Thicker arrows indicate the stiffer axis. (e)-(g) Box plots comparing aggregated *κ_X_* and *κ_Y_* values between treatments and T1C concentrations in (e) cell-free hydrogels and around (f) DFs and (g) MDAs. ^***^ *p* ≪ 0.01 and ^**^ *p* < 0.05 for (e)–(g).

ECM mechanical stiffness (*κ*) was measured using optical tweezers AMR [Fig. 1(a)]^10^ *κ* was measured around each cell along both horizontal (*X*′) and vertical (*Y*′) axes of the image field-of-view. Cells rarely align with the *X'* and *Y'* axes, and so stiffness was projected onto two new axes aligning with the long (*X*) and short (*Y*) axis of each cell, with the origin at the cell centroid [Figs. 1(b) and 1(c)]. Stiffnesses in this new coordinate system are denoted as *κ_X_* and *κ_Y_*.

### A. Effects of the ECM concentration and treatments on T1C hydrogel stiffness

Stiffness distributions are plotted in Figs. 1(e)–1(g). These plots aggregate *κ_X_* and *κ_Y_* for each probed bead. The aggregate stiffnesses are referred to as *κ*. Cell-free T1C hydrogels having an initial concentration of 1.0 mg/ml (1.0T1C) or 1.5 mg/ml (1.5T1C) were probed [Fig. 1(e) and supplementary material, Table I]. Stiffnesses of the gels were investigated 24 h after sample preparation and addition of treatment media. Treatment media included Dulbecco's Modified Eagle's Medium (DMEM) supplemented with 25 mM glucose, 10 ng/ml TGF-β1, or 20 μM Y27632. *κ* of cell-free hydrogels in control (untreated) conditions increased with concentration (*p* ≪ 0.01; *n_beads_* = 136 for 1.0T1C and *n_beads_* = 127 for 1.5T1C). Treatment conditions did not significantly affect *κ* within 1.0T1C (*p* = 0.79*)* or 1.5T1C (*p* = 0.32) hydrogels.

Next, ECM stiffness around DFs and MDAs was measured at the two T1C concentrations and three treatment conditions. Statistical testing results are found in Table II of the supplementary material. *p* values smaller than 0.01 were reported as *p* ≪ 0.01 and *p* values larger than 0.99 were reported as *p* > 0.99. Figure 1(f) summarizes results for DFs. For 1.0T1C hydrogels, *κ* was greater in control and treated DF cultures as compared to cell-free hydrogels, with the exception of Y27632 treatment, which did not differ from the cell-free condition (*p >* 0.99). As compared to DF control conditions, treatment with Y27632, glucose, or TGF-β1 decreased (*p* ≪ 0.01), increased (*p* ≪ 0.01), or did not significantly change (*p* = 0.65) stiffness, respectively. For DFs in 1.5T1C hydrogels, significant differences in stiffness were not detected between all treatment and cell-free conditions (supplementary material, Table II). Differences in *κ* were not detected between paired treatment groups at the two T1C concentrations (except for Y27632), which was surprising given that κ in cell-free hydrogels increased with the T1C concentration.

For MDA cultures, *κ* in control and treatment groups at either T1C concentration was not significantly different from respective cell-free conditions, with the exception of Y27632 treatment in 1.5T1C hydrogels, for which *κ* decreased (*p* ≪ 0.01) [supplementary material, Table II and Fig. 1(g)]. For all treatments, *κ* increased with T1C concentration (*p* ≪ 0.01).

**FIG. 2.**
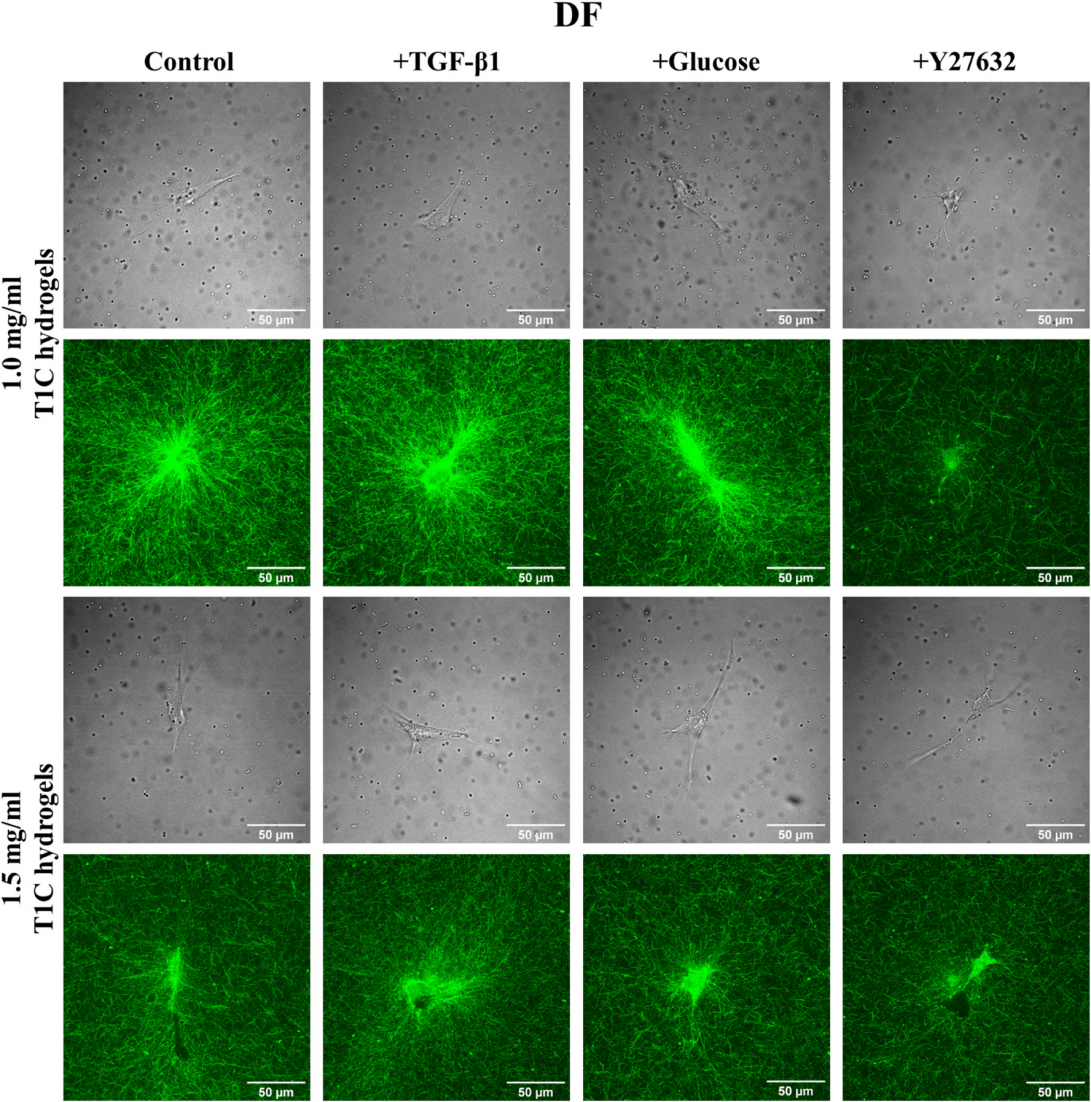
Brightfield and reflection confocal microscopy images of DFs embedded in T1C hydrogels with 2 *μ*m diameter silica microbeads.

**FIG. 3.**
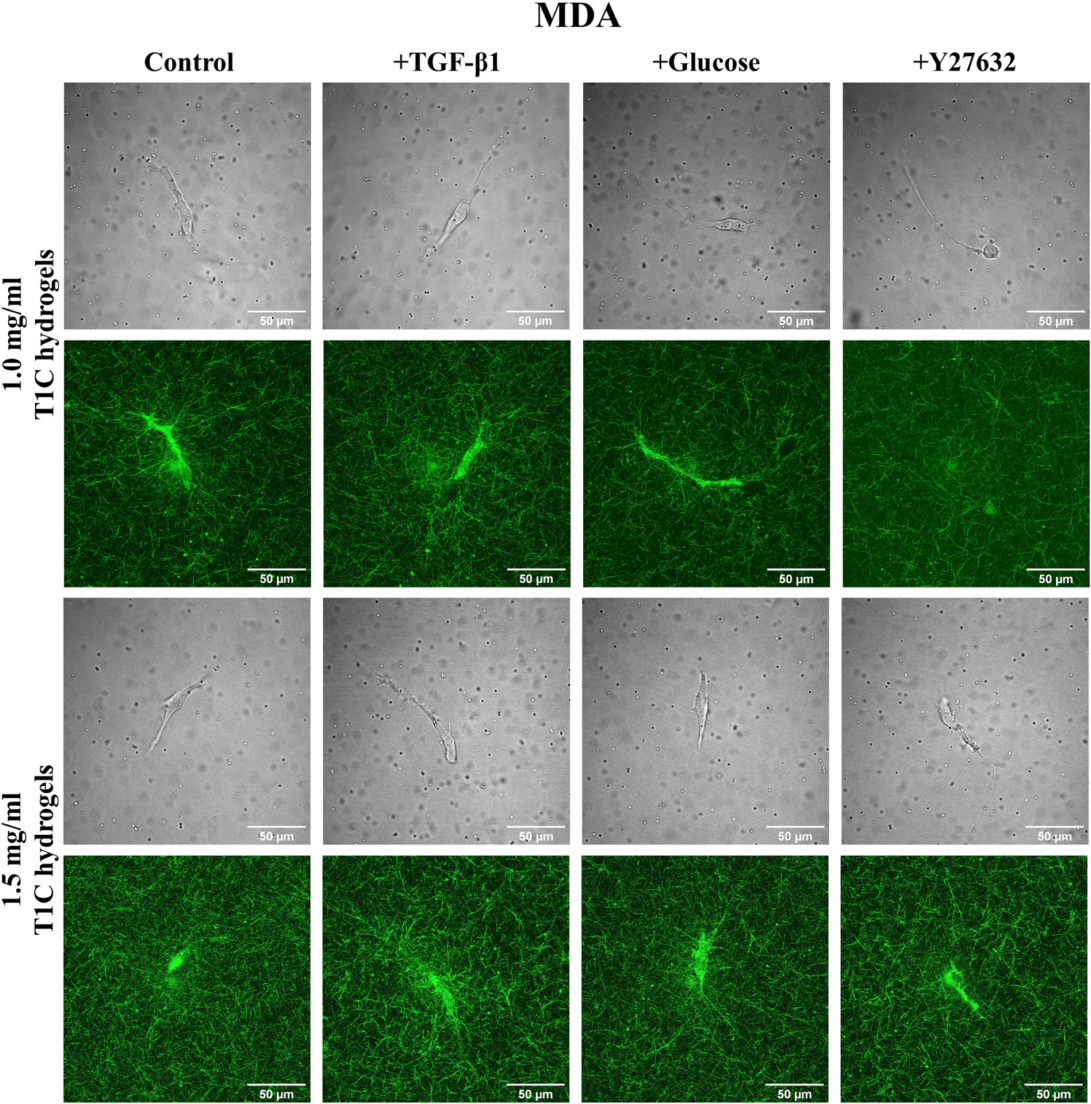
Brightfield and reflection confocal microscopy images of MDAs embedded in T1C hydrogels with 2 *μ*m diameter silica microbeads.

Figures 2 and 3 show that the two cell types take on different morphologies in the T1C concentration and treatment conditions. These morphologies can be described as elongated, and MDAs appear less contractile as compared to DFs. We investigated differences in stiffness anisotropy around these cells [Fig. 1(d) and supplementary material, Table III). In Fig. 1(d), the axis of greater stiffness is indicated by the thicker arrow. Differences between *κ_X_ and κ_Y_* were tested by the Wilcoxon signed rank test. Anisotropy was not detected in cell-free conditions. For both control DFs and MDAs, the stiffer axis transitioned from *X* to *Y* with increasing T1C concentration. Treatment conditions promoted distinct cell-line and T1C concentration-dependent trends in stiffness anisotropy.

### B. Peri-cellular stiffness distributions and anisotropy

**FIG. 4.**
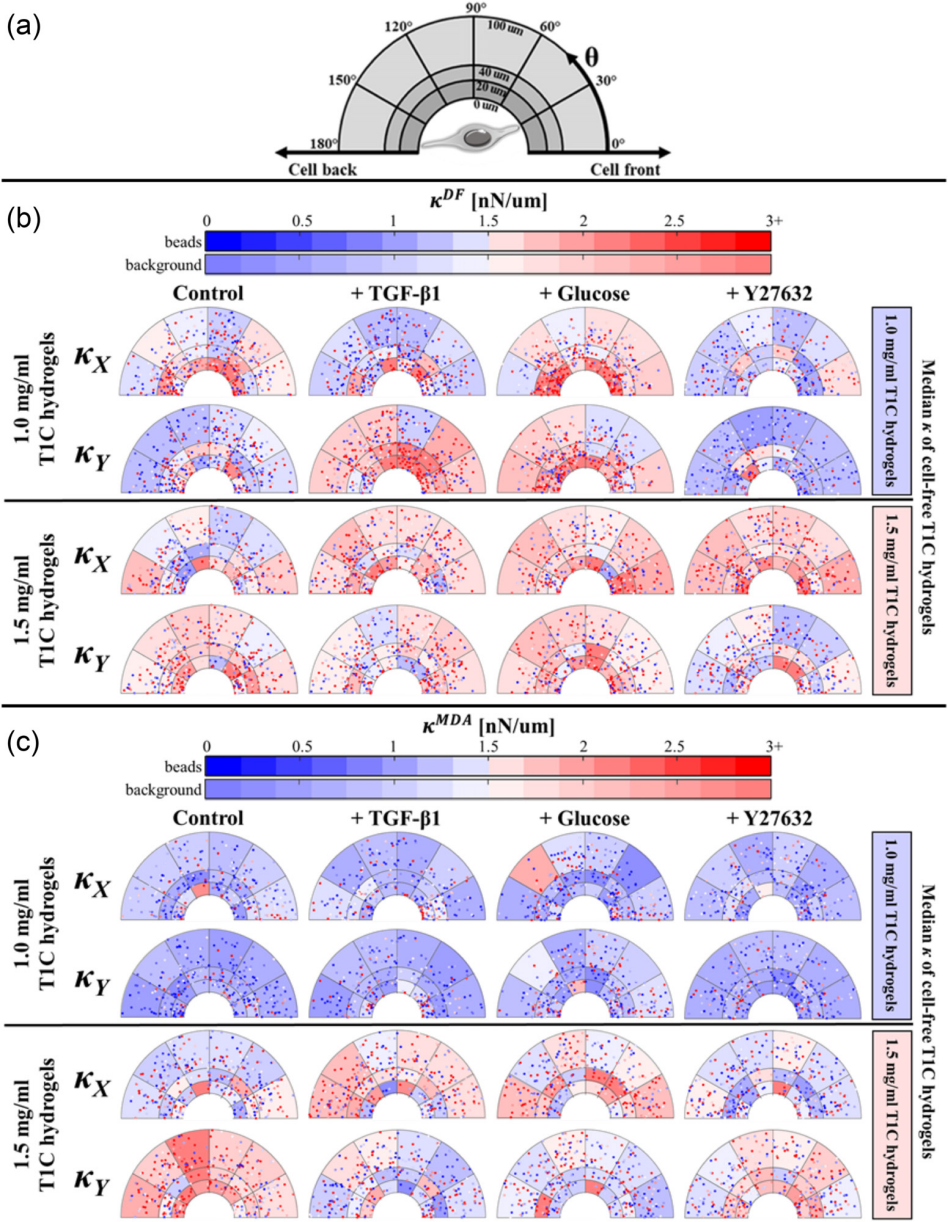
Hydrogel stiffness distributions for cell type, T1C concentration, and treatment groups. (a) Graphical representation of the coordinate system to discretize the ECM region around a cell. This coordinate system has the origin at the cell centroid with 0° pointing toward the leading edge of the cell. The coordinate system was folded upon itself along the *X* axis under the assumption of symmetry. Distribution of *κ_X_* and *κ_Y_* around (b) DFs and (c) MDAs in 1.0T1C and 1.5T1C. Bin background color is shaded according to the median value of *κ* in each bin (background color bar). Each data point is a single probed bead, color-coded for *κ* (beads color bar).

*κ_X_* and *κ_Y_* values in Fig. 1 are aggregated for all beads independent of location relative to their respective cell. We next examined the spatial distribution of *κ_X_* and *κ_Y_* relative to DFs and MDAs. Stiffness values were not normally distributed (*p* ≪ 0.01 by Kolmogorov–Smirnov testing) and compared using the Kruskal–Wallis test with Tukey–Kramer post-hoc testing at a significance level of 0.05. The results of the Tukey–Kramer tests are included in Tables IV—VI of the supplementary material. Our method for graphing *κ* stiffness distribution is illustrated in Fig. 4(a). Each probed bead is assigned two coordinates. The first coordinate is the shortest distance between the bead and cell profile. The second coordinate is the angular position *θ* relative to the *X* axis in the counterclockwise direction (with origin at the cell centroid). These two coordinates place each bead within one of the eighteen annular bins. The coordinate system was folded upon itself along the *X* axis, under the assumption of symmetry. By definition, beads having *θ*: 0–30° are located in the region of the cell leading edge (front), while beads having *θ*: 150–180° are located in the region of the trailing edge (back). The inner annulus from 0 to 20 *μ*m is considered the peri-cellular region, previously shown to stiffen around DFs cultured in T1C hydrogels.^10^ Figures 4(b) and 4(c) summarize the spatial distribution of *κ* surrounding DFs and MDAs at both T1C concentrations and all treatment groups. Each bin in Figs. 4(b) and 4(c) is shaded according to the median value of *κ* in that bin. Each point is a single probed bead and color-coded for *κ*.

### C. DFs increase local ECM stiffness of 1.0T1C but not 1.5T1C hydrogels

We first considered *κ_X_* under control conditions, where *κ_X_* is stiffness in the direction parallel to cell elongation. Peri-cellular (inner annulus) *κ_X_* values were comparable between T1C concentrations (*p* = 0.85, Table V of the supplementary material). This observation holds for the distal (outer annulus) region (*p* > 0.99, Table VI of the supplementary material). These results are surprising considering that stiffnesses of cell-free hydrogels increased with the T1C concentration [Fig. 1(e)]. For 1.0T1C hydrogels, *κ_X_* in the peri-cellular region was greater than that of cell free hydrogels (*p* ≪ 0.01), a finding that did not hold for the distal region (*p* = 0.29) and is suggestive of cell-mediated peri-cellular stiffening. For 1.5T1C hydrogels, *κ_X_* was not significantly different from *κ_X_* of cell free hydrogels in either the peri-cellular (*p* > 0.99) or distal region (*p* > 0.99).

*κ_Y_*, which is defined as stiffness in the direction perpendicular to cell elongation, showed T1C concentration dependency in the distal (*p* ≪ 0.01), but not the peri-cellular region (*p* > 0.99). Our results suggest that DFs in 1.0T1C, but not in 1.5T1C hydrogels, preferentially stiffen ECM in the direction of cell migration.

### D. MDAs do not increase local stiffness of T1C hydrogels

Control MDAs produced a relatively mild effect on their ECM [Fig. 4(c)]. Both *κ_X_* and *κ_Y_* increased with the T1C concentration (*p* ≪ 0.01, Table IV of the supplementary material). However, *κ_X_* was not different from cell-free conditions at either T1C concentration in both peri-cellular (supplementary material, Table V) and distal (supplementary material, Table VI) regions. Similarly, *κ_Y_* around MDAs did not differ from *κ_Y_* of cell-free hydrogels, with the exception of *κ_Y_* in the distal region in 1.0T1C, which was significantly reduced (*p* = 0.04) but only by 0.41 nN/*μ*m when comparing medians. Overall, MDAs did not alter their local stiffness to the extent observed for DFs.

Comparison between cell lines showed that DFs established higher ECM stiffness values than MDAs when cultured in 1.0T1C hydrogels (*p* ≪ 0.01). A difference in ECM stiffness was not detected between cell types cultured in 1.5T1C hydrogels nor were these stiffness values different from that of cell-free 1.5T1C hydrogels (supplementary material, Table II). The degree of stiffness anisotropy was similar for both cell lines and dependent on the T1C concentration. Both cell lines established an ECM that is stiffer in *X* than *Y* in 1.0T1C hydrogels (*p* ≪ 0.01) but stiffer in *Y* than *X* in 1.5T1C hydrogels [*p* ≪ 0.01, Fig. 1(d)].

### E. TGF-β1-treated cells establish different stiffness anisotropy than control cells

Treatment of DFs and MDAs with TGF-β1 was expected to promote peri-cellular stiffening based on the role of TGF-β1 in DF-stimulated collagen synthesis and EMT initiation in MDAs. For DFs in 1.0T1C hydrogels, the peri-cellular region stiffened along the *X* and *Y* directions when compared to cell-free conditions, but *κ_X_* and *κ_Y_* did not differ from control DF hydrogels (supplementary material, Table V). In the distal region, *κ_Y_* but not *κ_X_* was higher than respective stiffnesses of both cell-free and control DF hydrogels (supplementary material, Table VI). For DFs in 1.5T1C hydrogels, treatment with TGF-β1 did not result in significant changes in *κ_X_* or *κ_Y_* when compared to either cell-free hydrogels or control DF hydrogels in either region or direction (supplementary material, Tables V and VI).

For MDAs, a treatment effect was observed only in the distal region along the *Y* direction in 1.5T1C hydrogels. There, *κ_Y_* around cells was significantly lower than around control cells (*p* ≪ 0.01) but not different from cell-free hydrogels (*p* = 0.77, Table VI of the supplementary material).

Stiffness values around cells treated with TGF-β1 exhibited a reversed-directional bias in stiffness anisotropy. As shown above, both control DFs and control MDAs cultured in 1.0T1C hydrogels established an ECM stiffer in the *X* than *Y* direction (*κ_X_ > κ_Y_*). The opposite was true in 1.5T1C hydrogels (*κ_X_ < κ_Y_*). However, TGF-β1-treated DFs showed the reverse dependency on the T1C concentration such that *κ_X_ < κ_Y_* in 1.0T1C hydrogels and *κ_X_ > κ_Y_* in 1.5T1C hydrogels [*p* ≪ 0.01, Fig. 1(d)]. TGF-β1-treated MDAs established an ECM that is stiffer in the *X* direction at both T1C concentrations (*p* ≪ 0.01). These findings indicate that TGF-β1 promotes diverse and anisotropic patterns of *κ* around both cell lines, with stiffness anisotropy affected more than overall stiffness magnitude.

### F. Glucose-treated DFs but not MDAs establish stiffer and isotropic ECM

The addition of glucose was expected to increase *κ* around both cell lines, because glucose was previously reported to promote collagen synthesis by fibroblasts and invasiveness of MDAs.^4,24,28,32^ When looking at the overall *κ* (analyzing all beads probed up to 100 *μ*m from the cell) around DFs in 1.0T1C hydrogels, glucose treatment increased both *κ_X_* and *κ_Y_* as compared to control and cell-free hydrogels (supplementary material, Table IV). Stiffness around glucose-treated DFs in 1.5T1C hydrogels did not differ from that of cell-free hydrogels (supplementary material, Table IV). The overall stiffness around glucose-treated DFs was not significantly different between the two tested T1C concentrations (*p* > 0.99, Table IV of the supplementary material). Stiffness anisotropy was not detected at either T1C concentration [Fig. 1(d)].

MDAs treated with glucose established an isotropic *κ* distribution within 1.0T1C hydrogels [*p* = 0.29, Fig. 1(d)] resulting from a stiffening in the *Y* direction as compared to control cells (*p* ≪ 0.01, Table IV of the supplementary material). Stiffness around glucose-treated MDAs in 1.5T1C hydrogels was higher in the *X* direction (*p* ≪ 0.01), resulting from an increase in *κ_X_* (*p* = 0.04) and a decrease in *κ_Y_* (*p* ≪ 0.01) as compared to stiffness around control MDAs (supplementary material, Table IV). Interestingly, while *κ* around glucose-treated MDAs increased with the T1C concentration (*p* ≪ 0.01 in *X*, *p* = 0.03 in *Y*), *κ* did not differ significantly from the stiffness of corresponding cell-free hydrogels (supplementary material, Table IV). Consequently, glucose might have a less potent but more complex effect on MDAs than on DFs.

### G. Y27632-treated cells establish an ECM similar in stiffness to cell-free conditions

Y27632 treatment was selected to inhibit cell contractility and thus strain stiffening. Y27632 was previously shown by our group to reduce stiffness in the peri-cellular region of DFs to levels comparable to cell-free regions.^10^ In our current study, we first compared peri-cellular *κ* of DFs to the cell-free conditions. We found that peri-cellular *κ* at either T1C concentration did not differ from *κ* of cell-free hydrogels following Y27632 treatment (supplementary material, Table V). A similar result was found for distal regions, with the exception of *κ_Y_* around DFs within 1.5T1C hydrogels, which was lower than stiffness of cell-free hydrogels (*p* = 0.02, Table VI of the supplementary material). We next compared *κ* around control and Y27632-treated DFs. When considering all beads in all regions, Y27632 treatment resulted in an overall decrease in ECM stiffness around DFs cultured in 1.0T1C hydrogels when compared to control cells (supplementary material, Table IV). For DFs in 1.5T1C hydrogels, stiffness increased in the *X* direction (*p* = 0.01) but decreased in the *Y* direction (*p* = 0.01) as compared to control cells. However, when considering only the peri-cellular space around DFs treated with Y27632, only *κ_X_* in 1.0T1C hydrogels was different (reduced) from stiffness around control cells (supplementary material, Table V).

For MDAs treated with Y27632, stiffness did not differ significantly from cell-free conditions with the exception of *κ_X_* in 1.5T1C hydrogels, which was lower than stiffness of cell-free hydrogels (*p* = 0.01, Table IV of the supplementary material). We did not observe a difference in *κ_X_* or *κ_Y_* between control and Y27632-treated cells in either peri-cellular or distal regions (supplementary material, Tables V and VI), which was not surprising given the insignificant strain stiffening by control cells (stiffness around control MDAs was not significantly different from cell-free conditions, supplementary material, Table IV).

## III. DISCUSSION

Cell contractile forces were previously shown by our group to establish highly heterogeneous κ distributions around individual DFs with significant ECM stiffening in the peri-cellular region as compared to cell-free hydrogels.^10^ Here, we investigated κ distributions around DFs and invasive triple-negative breast cancer MDAs embedded at two different T1C concentrations. Both cell lines were previously reported to be highly migratory, yet phenotypically and morphologically different.^33,34^ Thus, we investigated if and how patterns of κ distribution differ between these two cell lines. In a first set of experiments, we simply cultured these cells in fibrous T1C hydrogels polymerized at 1.0 mg/ml and 1.5 mg/ml. While the concentration of T1C does increase by 50%, the absolute concentration difference is modest as compared to previously published experimental systems that used T1C hydrogels in the range of 0.5–4.0 mg/ml.^9,35–38^ DFs were found to be considerably more responsive to the change in concentration than the MDAs—and in some surprising ways. For example, stiffness in cell-free hydrogels increases with the T1C concentration (by 84%; comparing median values) as expected and verified by both AMR [Fig. 1(e)] and macrorheology.^9,39,40^ As replicated in previous work,^10^ we found DFs increase their local stiffness in 1.0T1C hydrogels as compared to cell-free conditions. Surprisingly, when cultured in 1.5T1C hydrogels, these cells “chose” not to stiffen their local ECM values. In fact, when considering all probed regions and both probed axes, there are no significant differences in stiffness between the groups of control DFs in 1.5T1C hydrogels, control DFs in 1.0T1C hydrogels, and cell-free 1.5T1C hydrogels [Fig 1(f)].

A closer examination of the peri-cellular region of DFs shows differential stiffness with the T1C concentration, if considering local anisotropies. In cell-populated T1C hydrogels, anisotropy of collagen fiber alignment is attributed to cell-induced alignment of the matrix fibers during migration, contraction, or enhanced long range stress signaling between neighboring cells.^41–43^ While stiffness along the axis perpendicular to cell elongation is equivalent between T1C concentrations (*p >* 0.99), stiffness parallel to migration is larger in 1.0T1C hydrogels than in 1.5T1C hydrogels (*p* ≪ 0.01). Overall, our findings show that DFs in 1.0T1C hydrogels respond to and considerably increase local ECM stiffness as compared to values in the cell-free condition. ECM stiffening is also much more prominent in the peri-cellular region than in the distal region (*p* ≪ 0.01 in the *X* direction and *p* = 0.02 in the *Y* direction). This finding is in agreement with the previous studies showing that ECM accumulation decreases as a function of distance to the cell.^44^ By contrast, the DF cells do little to change their local stiffness landscape in 1.5T1C hydrogels, in which ECM stiffness is comparable between peri-cellular and distal regions (*p* = 0.74 in the *X* direction and *p* = 0.14 in the *Y* direction). This differential behavior indicates DFs might intrinsically prefer certain stiffness levels or have a set point. While such an effect of the T1C concentration on peri-cellular stiffness has not been reported previously, it has been shown that the contractility of human fetal lung fibroblasts, human aortic adventitial fibroblasts, bone marrow stromal cells, and DFs decreases with the T1C concentration.^38,45–48^

MDAs, the other migratory cell line under investigation, do not behave similarly to DFs. MDAs do not appear to significantly alter local stiffness values when comparing treatment and cell-free conditions, but stiffness did increase with the T1C concentration. As compared to DFs, MDAs exhibit a smaller extent of ECM stiffening with less matrix reorganization (Fig. 3), which is in agreement with past studies.^49^ Studies also indicate that MDAs can employ different strategies and modes of migration to adapt to ECMs of varying stiffness, which promotes invasion mechanisms and cancer metastasis.^50^ Consequently, unlike DFs that utilize pseudopodia-based migration, MDAs might favor protrusion-based amoeboidal migration in our experimental conditions. Such migration is usually observed in migratory cells exhibiting a lesser degree of cell contractility and adhesion.^33,49,51^ Amoeboidal migration should thus result in a lesser degree of strain stiffening, as our results show. Furthermore, we expected that TGF-β1 and glucose, known to increase MDA invasiveness, would invoke an increase in MDA-mediated matrix stiffness. Such stiffening was not observed, but changes in anisotropy were observed [Fig. 4(c)]. This finding is corroborated by previous work showing the invasive potential of breast cancer cells was more correlated with the directionality of the cell contractility than magnitude of cell traction forces.^52^ Of note, a finite element analysis of principal matrix stiffness around MDAs in 1.2T1C hydrogels predicts a decrease in κ close to cells,^39^ as we observed. However, another study used AMR with larger microbeads (4.5 *μ*m) and found MDAs establish long range stiffening in 1.5T1C hydrogels,^53^ which is in disagreement with both the FEM model and our own results, yet might be observed in mesenchymal (as opposed to amoeboidal) MDAs.^49^ In support of our findings, confocal reflection images of MDAs (Fig. 3) demonstrate that MDAs do not significantly contract their local ECM and, as a result, the values of κ are lower than around DFs, which visibly stiffen and contract their surrounding ECM (Fig. 2).

Here, we also test three different treatments, which were expected to alter κ levels. TGF-β1 and glucose were expected to stiffen the local ECM of both DFs and MDAs because of their effect on increasing contractility of DFs^12,28,38^ and invasiveness of MDAs.^13,21^ As anticipated, when analyzing all probed beads, treatment with glucose does lead to an overall stiffer ECM near DFs. Interestingly, glucose treatment also promotes isotropic stiffening at both concentrations, with no preference to the axis of migration. In contrast, treatment with TGF-β1 did not result in prominent stiffening, which is in agreement with past studies that found a limited effect of TGF-β1 on the contractility of fibroblasts 24 h after treatment^54^ or when cells were seeded at low density.^38^ However, TGF-β1 did alter the extent of stiffness anisotropy around DFs [Fig. 4(b)]. In 1.0T1C hydrogels, control DFs establish local anisotropy and larger stiffness in the direction of migration, but this trend reverses with TGF-β1 treatment. The opposite relationship is observed for 1.5T1C hydrogels. Our results may be explained in part by findings that TGF-β1 promotes actin reorganization and stress fiber formation,^55^ which might manifest as a change in stiffness anisotropy due to strain stiffening. Furthermore, our finding that overall stiffness was not increased with TGF-β1 treatment may not hold over longer culture times as supported by studies showing that effects of TGF-β1 on contractility continue to increase beyond our 24 h time point.^38,54^

Surprisingly, unlike DFs, the addition of either glucose or TGF-β1 to MDAs does not affect overall stiffness values but does alter anisotropy [Fig. 1(g)]. For control cells, ECM stiffness is higher along the axis of migration in 1.0T1C hydrogels but in 1.5T1C hydrogels, anisotropy patterns are switched so that ECM is up to 2.2 times stiffer orthogonal to cell migration. By contrast, TGF-β1 treatment results in higher stiffness along the axis of migration at both T1C concentrations. This effect of TGF-β1 on stiffness anisotropy can be corroborated by past studies, which attributed higher motility, deformability, and a more amoeboidal phenotype of MDAs to TGF-β1 treatment.^33,56^ In the case of glucose treatment, this anisotropy favoring the direction of cell migration became more pronounced with the T1C concentration even though overall stiffness did not significantly change as compared to cell-free conditions. This lack of overall stiffening is consistent with studies showing that the degree of glycolysis within MDAs did not change significantly as glucose in the media increased from 25 to 50 mM glucose^57^ or when the T1C concentration increased from 1.2 to 3.0 mg/ml.^9^ Our culture conditions overlap those of these studies, and collectively our findings suggest that to better elucidate the effect of glucose on peri-cellular stiffness, a wider range of glucose and T1C concentrations altering cell metabolic activity should be investigated. Nonetheless, while neither treatment significantly alters overall stiffness [Fig. 1(g)], accounting for spatial information of probed beads and axis of measurements elucidates more complex treatment effects [Fig. 4(c)].

Finally, the addition of Y27632 was expected to lower κ levels, as previously reported by our group.^10^ Y27632-induced reduction in cell contractility was previously described for both DFs and MDAs.^29,58^ Here, we find that Y27632 treatment significantly lowers overall *κ* around DFs as compared to control cells at both tested T1C concentrations. However, ECM stiffening as compared to cell-free conditions can still be observed in the peri-cellular region, which is in agreement with past studies^10,44^ and may be indicative of ECM remodeling. Previous studies have shown that Y27632 does not fully prevent local strain stiffening around highly contractile DFs, which can still deposit T1C and cross-link existing ECM to an extent comparable with control cells in the peri-cellular region.^44^ Studies on rat embryo fibroblasts in T1C hydrogels have also shown only 52% reduction in cell contractility 27 h after adding Y27632 (10 μM).^59^ While treatment of MDAs with Y27632 was shown to reduce ROCK activity by ∼50%,^29^ in our study, treatment effects on MDAs were mild with respect to stiffness. Stiffness was comparable to, or even lower than, that of cell-free conditions, suggesting that MDAs are still capable of proteolysis induced by matrix metalloproteinase activity.^10^ The relative insensitivity of MDAs to Y27632 can be explained in part by the observation that these cells did not stiffen their ECM as compared to cell-free conditions. In other words, there may not be very much strain stiffening to alleviate.

In summary, AMR measurements reveal highly heterogeneous patterns of ECM stiffness around individual cells. Heterogeneities in local ECM properties have been widely reported and are also observed in cell-free hydrogels. Heterogeneities in stiffness to some extent can be indicative of differences in local fiber mesh architecture as well as properties of the collagen fibers. However, given that all cells were cultured in similar T1C hydrogels polymerized at either 1.0 or 1.5 mg/ml, we can assume that differences in local stiffness and isotropy are primarily attributed to cell-induced changes. Cells were previously shown to dynamically alter local ECM density by incessant interplay of ECM compaction and cross-link unbinding.^60^ While we are unable to identify whether probed beads are attached to fibers undergoing compaction or cross-link unbinding, optical tweezers AMR is sensitive to changes in stiffness of the local mesh ensemble of these fibers. Compared to other techniques for quantifying cell-induced changes in ECM properties, AMR is not as invasive as atomic force microscopy or as destructive as laser ablation.^61^ One limitation to our microrheology method is the technical inability to align the axes of bead oscillation with the cell and reliance on projecting the *X* and *Y* axes. Future experiments will aim to align these axes and eliminate the potential errors associated with such projection. Nonetheless, we found κ levels around both cell lines to be dependent on tested treatment and axis of measurement, yet to different extents, with MDAs establishing overall lower κ than DFs. Our results not only illustrate how cells can both adapt and modify their local ECM in response to different factors but also highlight shortcomings of bulk stiffness measurements. Bulk rheology obscures microscopic understanding of treatment effects, which show notable heterogeneity by microrheology. Notably, despite an increase in bulk (cell-free) stiffness with an increase in the T1C concentration, the peri-cellular stiffness around DFs was actually found to be comparable between T1C concentrations, and in some instances, stiffness decreased with the T1C concentration. Additional studies are required to further investigate this relationship between initial and final ECM stiffness and to investigate if particular cell types remodel their ECM to achieve a stiffness set point.

## IV. METHODS

Ethics approval is not required for the methods of this study.

### A. Cell culture

Normal human dermal fibroblast (DF) cells were cultured in Dulbecco's Modified Eagle's Medium (DMEM) with low glucose, L-glutamate, and sodium pyruvate (ThermoFisher) with 10% Fetal Bovine Serum (FBS) (Gibco) and 1% penicillin streptomycin (Gibco). All cells were used prior to passage 8.

Human breast cancer cells MDA-MB-231 (MDAs) were cultured in DMEM with high glucose, L-glutamate, and sodium pyruvate (ThermoFisher) with 10% FBS (Gibco) and 1% penicillin streptomycin (Gibco).

### B. Collagen hydrogel preparation

Cells were embedded in type I collagen due to its abundance in the natural ECM of MDAs and DFs. Hydrogels at 1.0 and 1.5 mg/ml concentrations were prepared using type I rat tail telocollagen (Advanced Biomatrix), 10X Phosphate-Buffered Saline (PBS) with added calcium and magnesium (ThermoFisher), 10X DMEM (Sigma), 10X reconstitution buffer prepared as described by Doyle,^62^ 1 N NaOH (ThermoFisher), 2 *μ*m carboxylated silica microbeads (0.8 mg/ml, Bangs Laboratories), and cells (50 k/ml) in 35 mm glass bottom dishes (MatTek). Each hydrogel was allowed to polymerize for 30 min in a standard tissue culture incubator at 37 °C and 5% CO_2_ prior to adding 2 ml of media. Media was supplemented with 25 mM HEPES (ThermoFisher) and different treatments: 10 ng/ml TGF-β1 (PeproTech), 25 mM glucose (Sigma), or 20 *μ*M Y27632 (PeproTech). Gels were incubated for 24 h prior to AMR measurements.

### C. Active microrheology (AMR)

The AMR system used in our laboratory is presented in Fig. 1 of the supplementary material. It incorporates a continuous-wave fiber laser with an emission at 1064 nm (YLR-5–1064-LP, IPG Photonics). The power of the expanded beam entering the objective lens is ∼240 mW. The trapping beam is oscillated by the movement of a pair of galvanometer mirrors (GVS012, ThorLabs), which are placed conjugate to the back focal plane of the microscope objective lens. The trapping beam is sampled just beyond the galvanometer mirrors using a beam splitter (BSF20-C, ThorLabs), which allows the beam position to be recorded by a quadrant photodiode (trapQPD, 2903, Newport). The detection beam is generated by a single mode fiber-pigtailed laser (LP785-SF100, ThorLabs) with emission at 785 nm and a power of 22 mW. Both beams are coaligned by a long-pass dichroic beam splitter (FF875-Di01, Semrock) and enter the white light path of an IX81 inverted microscope (Olympus). As described in Ref. 10, the microscope in our laboratory is equipped with a short pass dichroic beam splitting mirror (ET750SP-2P8, Chroma Technologies) below the microscope objective lens where the Zero Drift Compensation package (Olympus) was designed to fit. The beam splitting mirror passes visible light for confocal and brightfield microscopy and reflects both trapping and detection beams into the sample. Light is focused by a high numerical aperture microscope objective lens (60x-oil PlanApo TIRFM 1.45 NA, Olympus). The focus height for both beams was adjusted to be approximately equal. Light from the sample is then back reflected and the detection beam is separated from the trapping beam by the long pass dichroic beam splitter (D2). Then, a 45/55 pellicle beam splitter (CM1-BP145B2, ThorLabs) transmits the light toward the detection beam quadrant photodiode (detQPD, 2901, Newport).

During the sinusoidal oscillations of the trapping beam $\left(x_{T}\right)$, the position of the bead $\left(x_{B}\right)$ in the direction of bead oscillation is recorded by the detQPD, which provides analog signals proportional to the displacement of the bead. Ignoring any small off axis movements of the bead, we can treat the experiment as a one-dimensional problem. The applied optical force acting in the direction of bead oscillation (either the *X*′ or *Y*′ direction) is expressed by

$$f(t)=k_{T}\;(x_{T}(t)-x_{B}(t)),$$ (1)

where $k_{T}$ corresponds to the trap stiffness that is calculated during calibration.

The local complex material response is described in the Fourier space as

$$\alpha^{*}(\omega )=X_{B}(\omega )/(F(\omega )-k_{T}X_{B}(\omega )),$$ (2)

where $X_{B}$ and F are the Fourier transforms of $x_{B}$ and f. Stiffness *κ* is then defined as the real component of 1/$\alpha^{*}(\omega )$. Under the assumption of a continuum, the complex shear modulus $G^{*}(\omega )$ can be defined as

$$G^{*}(\omega )=1/6\pi r\alpha^{*}(\omega ),$$ (3)

where *r* corresponds to the radius of the bead (1 *μ*m). *G′* and *G″* for DFs and MDAs are included in Figs. 4 and 5 of the supplementary material, respectively, and the data show the hydrogels are viscoelastic having storage modulus greater in magnitude than loss modulus, at the probed frequency.

Our AMR system is controlled by custom software developed in our laboratory and described in Ref. 10. It allows for precise stage positioning to center a microbead in the optical trap. Each bead was probed by the optical trap oscillating at frequencies of 20 (supplementary material, Fig. 2), 50, and 100 Hz (supplementary material, Fig. 3) in both horizontal (*X*′) and vertical (*Y*′) axes. 50 Hz measurements were repeated twice. Each bead was probed for 5 s at each frequency for a total of 40 s measurement time. In all hydrogels, probed beads were only treated as outliers and discarded if, during the AMR measurements, bead centering was observed to be inaccurate or stiffness values either were negative or exceeded 60 nN/μm.

For stiffness measurements, collagen gels were incubated on the microscope stage using a Culture Dish Incubator (Warner Instruments) and an Objective Warmer (Warner Instruments) and allowed 20 min to equilibrate to 37 °C prior to AMR. At least 30 beads in close proximity to each cell were analyzed. The AMR system and stage incubator were turned on at least 1.5 h prior to system calibration and measurements on collagen gels to alleviate effects of alignment drift as the system comes to temperature.

*κ* was measured around 10 cells per condition in both 1.0 and 1.5 mg/ml T1C hydrogels. Cells included in the study had to meet the following criteria: (a) cells had to be predominantly in focus in the *XY* plane; (b) cells had to be isolated from other cells by few camera fields-of-views; and (c) cells had to exhibit an elongated morphology, characteristic of both cell lines. Up to two cells were studied per hydrogel, with probing at least 30 beads located within an in-focus image area bounded by 100 *μ*m from the cell surface (in plane) and ±6 *μ*m in depth. For each bead, *κ* was measured along both the *X*′ and *Y*′ axes with respect to the image field-of-view. *κ* measurements were then projected onto a new set of axes (*X* and *Y*) by rotating the *κ* values by the cell orientation angle, as described in Sec. IV E.

### D. System validation and calibration

The AMR system was calibrated in water prior to each experiment. A frequency sweep was conducted for at least 3 beads oscillated first in the *X′* (horizontal) direction and then in the *Y′* (vertical) direction, as previously described in Ref. 10. Briefly, a bead is trapped by both lasers and brought to a height of 35 *μ*m above the glass. With great care, both lasers are co-aligned in *X′*, *Y′*, and *Z′* and individually centered on the bead. Next, the detQPD and trapQPD are positioned by a 2-axis mount until mean voltages have a value of zero. Brownian motion of each bead is recorded for 30 s and analyzed using the power spectrum method.^63^ Trap stiffness $k_{t}$ was found separately in the *X′* and *Y′* directions. Afterwards, a position sweep of the bead was used to obtain the detQPD voltage-to-bead displacement factor β for each axis. Average $k_{t}$ and β values specific for each axis of oscillation were then used for AMR measurements in water (for calibration validation) as well as T1C hydrogels.

The viscosity η of water is known to be equal to 0.69 mPa·s at 37 °C.^64^ AMR measurements at f= [10 20 50 75 100] Hz were conducted in water samples maintained at 37 °C. Experimentally determined viscosity values were calculated as η=G″/2πf and compared to the theoretical value (0.69 mPa·s). Based on 8 separate calibrations at 50 Hz, each with at least 3 different beads, η values differed from the theoretical value, on average, by 3.95% in the *X′* direction and 5.64% in the *Y′* direction.

Errors due to automated motorized-stage and objective lens positioning were characterized. Beads were suspended in 1.0 mg/ml T1C hydrogels maintained at 37 °C. In a first experiment, we selected 29 beads across several fields of view and the automated system centered each bead to conduct AMR measurements. The purpose of this experiment was to determine errors in *κ* due to stage/objective positioning. In a second experiment, the system positioned each of the 32 beads and made 5 repeated measurements without moving the stage. The purpose of this experiment was to determine errors due to the system exclusive of bead positioning. For the first experiment, measurement error in *κ*, defined as standard deviation/mean · 100%, was equal to 7.52% and 6.56% along the *X′* and *Y′* directions, respectively. For the second experiment, error was 4.03% and 4.56% in the *X′* and *Y′* directions, respectively.

Frequency AMR sweeps at 10, 20, 50, 75, and 100 Hz indicated an increase in stiffness with frequency of bead oscillations in both *X′* (30 beads, *p* ≪ 0.01) and *Y′* directions (29 beads, *p* ≪ 0.01), based on the Friedman test for repeated measures. These findings are in agreement with the widely reported frequency effect on stiffness levels in microrheological studies.^63,65,66^

### E. Cell orientation assessment

AMR measurements around each cell were divided into several fields-of-view. Brightfield images of the cells were taken before AMR measurements on each field-of-view using an EO-4010 Monochrome USB 3.0 Camera (Edmund Optics) incorporated in our AMR system. Brightfield images were then processed in MATLAB (The MathWorks Inc.) using the image processing toolbox. The cell morphology was quantified by manual tracing, and MATLAB functions computed the angle of cell orientation, position of the cell centroid, and long and short axes of the cell per field-of-view. Furthermore, the spatial location of each bead with respect to the position and orientation of each cell was calculated in MATLAB. The shortest distance between the bead and cell profile was found by comparing pixel coordinates of each bead probed in a given field-of-view with the pixel coordinates of the manually traced cell shape. The distance in pixels was converted into micrometers. Angular position *θ* from −180 to 180° relative to the *X* axis was found by calculating the angle between the pixel position of the bead and centroid of the cell and subtracting the angle of cell orientation. The coordinate system was then folded upon itself along the *X* axis, under the assumption of symmetry; thus, *θ* ranged from 0 to 180°.

After AMR measurements, brightfield and reflection confocal images of the cells were acquired every 30 s for an additional 10 min. Confocal images were acquired using the 488 nm laser of the Fluoview 1200 laser scanning confocal microscope (Olympus) integrated into the AMR system. Analysis of the image series identifies the direction of cell migration and, consequently, the leading and trailing edge of the cells. If the direction of cell migration was not obvious during these 10 min, then brightfield images were compared with brightfield images taken at the start of the AMR measurements, which typically occurred 30–40 min earlier. Beads distal to the cell served as fiducial markers for cell migration.

### F. Statistical analyses

Data were not normally distributed (*p* ≪ 0.01, Kolmogorov-Smirnov test) necessitating non-parametric statistical analyses. The Wilcoxon test was used for the comparison of correlated measurements in *X* and *Y* directions [Fig. 1(d)] and the Kruskal–Wallis test for the comparison of multiple groups, with the post-hoc Tukey–Kramer test to compare specific groups (supplementary material, Tables II and IV—VI). Statistical testing was conducted at a significance level of 0.05. *p* values smaller than 0.01 were reported as *p* ≪ 0.01 and *p* values larger than 0.99 were reported as *p* > 0.99.

## SUPPLEMENTARY MATERIAL

See the supplementary material for additional experimental data and information.

## Data Availability

The data that support the findings of this study are available from the corresponding author upon reasonable request.
